# Erectile Function, Sexual Satisfaction, and Cognitive Decline in Men from Midlife to Old Age

**DOI:** 10.1093/geroni/igab046.3590

**Published:** 2021-12-17

**Authors:** Riki Slayday, Tyler Bell, Michael Lyons, William Kremen, Carol Franz

**Affiliations:** 1 The Pennsylvania State University, State College, Pennsylvania, United States; 2 University of California San Diego, La Jolla, California, United States; 3 Boston University, Boston, Massachusetts, United States

## Abstract

We investigated how changes in erectile function and sexual satisfaction relate to cognitive decline in men from midlife into early old age. This is a major transitional period for increased incidence of erectile function and for cognitive decline. We examined 833 men from the Vietnam Era Twin Study of Aging whose mean ages were 56, 61, and 68 at the time of assessment. Erectile function and sexual satisfaction were measured using scores from the International Index of Erectile Function. Individuals with erectile dysfunction at baseline were excluded. Cognitive performance was measured using factor scores for separate domains of episodic memory, executive function, and processing speed. We tested linear mixed models hierarchically adjusted for demographics, sexual activity, as well as physical and mental health confounders to examine how changes in erectile function and sexual satisfaction related to changes in cognitive function. Declines in erectile function were associated with declines in episodic memory (p=.004, d=.25), while declines in sexual satisfaction were associated with declines in processing speed (p=.006, d=.19). Decreasing erectile function and sexual satisfaction may be indicative of individuals also likely to be facing cognitive decline. Possible mechanisms accounting for these changes may include white matter microvascular disease and/or various lifestyle influences. Discussing and tracking sexual health with middle aged men may be a crucial step in identifying those likely to face cognitive decline.

